# Air Pollution–Related Prothrombotic Changes in Persons with Diabetes

**DOI:** 10.1289/ehp.0900942

**Published:** 2009-10-22

**Authors:** Lotte Jacobs, Jan Emmerechts, Chantal Mathieu, Marc F. Hoylaerts, Frans Fierens, Peter H. Hoet, Benoit Nemery, Tim S. Nawrot

**Affiliations:** 1 Occupational and Environmental Medicine, Unit of Lung Toxicology; 2 Center for Molecular and Vascular Biology and; 3 Department of Endocrinology, Katholique Universiteit Leuven, Leuven, Belgium; 4 Belgian interregional Environment Agency, Brussels, Belgium; 5 Centre for Environmental Sciences, Hasselt University, Diepenbeek, Belgium

**Keywords:** air pollution, diabetes, platelet function, systemic inflammation

## Abstract

**Background:**

Population studies suggest that persons with diabetes are more sensitive to the effects of particulate matter (PM) air pollution. However, the biological mechanisms of a possible prothrombotic effect underlying this enhanced susceptibility remain largely unknown.

**Objective:**

We hypothesized that exposure to PM causes prothrombotic changes in persons with diabetes, possibly via systemic inflammation.

**Methods:**

Our study included 137 nonsmoking adults with diabetes who were outpatients at the University Hospital Leuven. Recent exposure (2 hr before examination) to ambient PM was measured at the entrance of the hospital. Individual chronic exposure to PM was assessed by measuring the area occupied by carbon in airway macrophages obtained by sputum induction. Platelet function was measured *ex vivo* with the PFA-100 platelet function analyzer, which simulates a damaged blood vessel; we analyzed the function of platelets in primary hemostasis under high shear conditions. Total and differential blood leukocytes were counted.

**Results:**

Independent of antiplatelet medication, an interquartile range (IQR) increase of 39.2 μg/m^3^ in PM_10_ (PM with aerodynamic diameter ≤ 10 μm) concentration measured 2 hr before the clinical examination (recent exposure) was associated with a decrease of 21.1 sec [95% confidence interval (CI), − 35.3 to − 6.8] in the PFA-100 closure time (i.e., increased platelet activation) and an increase in blood leukocytes of 512 per microliter of blood (95% CI, 45.2–979). Each area increase of 0.25 μm^2^ (IQR) in carbon load of airway macrophages (chronic exposure) was associated with an increase of 687 leukocytes per microliter of blood (95% CI, 224–1,150).

**Conclusions:**

A relevant increase in recent PM exposure was associated with a change in platelet function toward a greater prothrombotic tendency. The magnitude of the change was about two-thirds (in the opposite direction) of the average effect of antiplatelet medication. Diabetic patients showed evidence of proinflammatory response to both recent and chronic exposure to PM air pollution.

Urban pollution, especially by particulate matter (PM), contributes to respiratory and cardiovascular morbidity and mortality (Dominici et al. 2006; Maitre et al. 2006; Pope et al. 2002; Simkhovich et al. 2008). To a large extent, the increase in mortality linked to PM ≤ 10 μm in aerodynamic diameter (PM_10_) is attributable to cardiovascular diseases (Maitre et al. 2006; Nawrot et al. 2006). Persons with diabetes who also have cardiovascular disease appear to be more sensitive to the effects of air pollution on daily mortality (Goldberg et al. 2006). Zanobetti and Schwartz (2002) also found stronger associations between increased levels of PM and hospitalizations for heart disease among those who had diabetes compared with those who did not. The risk of coronary heart disease, stroke, and peripheral arterial disease is increased in persons with diabetes (Brand et al. 1989; Stamler et al. 1993). Both atherosclerosis and thrombosis appear to contribute to this increased cardiovascular risk (Colwell 1997; Colwell and Nesto 2003). Therefore, research on environmental factors that may aggravate the disease, and on the mechanisms underlying this, has substantial public health relevance.

One of the problems in epidemiologic studies is estimating individual exposure to PM. In this study, the chronic exposure to PM was estimated at an individual level by determining the carbon load of airway macrophages, as described by Kulkarni et al. (2006). This approach is based on the fact that airway macrophages are the primary phagocytotic cells of inhaled PM. The amount of carbonaceous PM extracted from the lung at autopsy reflects the chronic exposure to PM (Brauer et al. 2001). We hypothesized that exposure to PM causes prothrombotic changes in persons with diabetes, possibly via systemic inflammation.

## Materials and Methods

### Study Population

Persons with both type 1 and type 2 diabetes were consecutively recruited from the diabetes outpatient clinic at the University Hospital Leuven. This clinic is a dedicated clinic for the routine 3- to 6-month follow-up of patients with diabetes. All patients were invited to participate on days when the investigator was present. They were included if they were ≥ 18 years of age and were nonsmokers. The study was carried out on different days from February 2007 through September 2008. On the study day, patients completed a questionnaire to obtain information on age, occupation, socioeconomic status, exposure to environmental tobacco smoke, alcohol use, use of medication, use of oral contraception, menopausal status, place of residence, and means of transportation to the hospital. Socioeconomic status was coded and condensed into a scale with scores ranging from 1 to 3. Use of antiplatelet medication was coded as use of either none or one or more of the following substances: acetylsalicylic acid, clopidogrel, ticlopidine, or dipyridamole. Distances from the home address to major roads were calculated through geocoding. Living close to a major road was defined as living within 100 m of an N-road or an E-road (Hoffmann et al. 2007).

**Figure 1:**
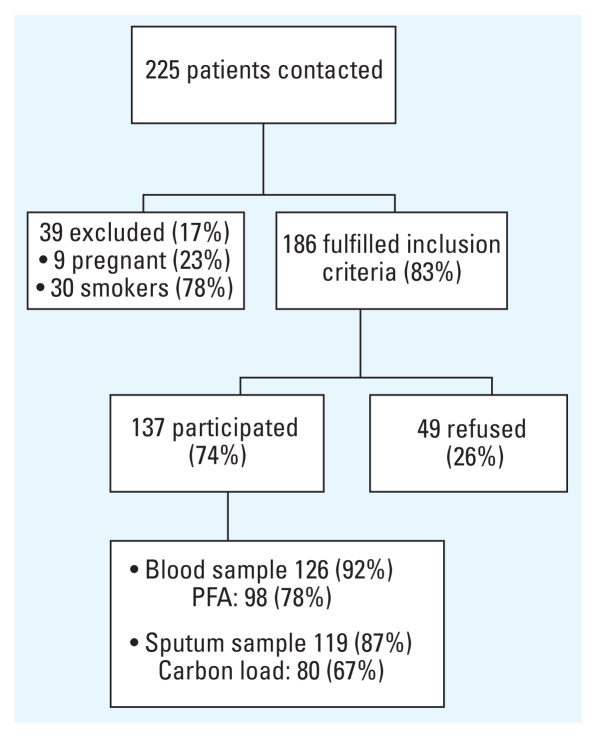
Flowchart of study population, consecutively recruited from the diabetes outpatient clinic at the University Hospital Leuven. Patients were included if they were ≥ 18 years of age and nonsmokers; 63 subjects had data on both platelet function (measured by PFA-100 platelet function analyzer) and carbon load of airway macrophages.

Of the 186 recruited subjects, 137 (74%) took part in the examination (Figure 1). The 49 patients that did not participate had the same age and sex distribution as the 137 participants. Sufficient numbers of airway macrophages to assess the area occupied by carbon were obtained from 80 of the 119 patients (18 of the 137 patients failed to produce sputum). A blood sample could not be obtained from 11 subjects, and platelet function analysis was not successful in 28 subjects. Ultimately, 63 subjects had data for both the carbon load of airway macrophages and the platelet function analysis.

The Ethics Review Board of the Medical Faculty of the University of Leuven approved the study. Participants gave informed consent at recruitment.

### Exposure Assessment

#### Ambient PM

##### Recent exposure

A portable laser-operated aerosol mass analyzer (Aerocet 531; Met One Instruments Inc., Grants Pass, OR, USA) was used to measure PM_2.5_ (PM with an aerodynamic diameter ≤ 2.5 μm) and PM_10_ concentrations 2 hr before the patient’s participation in the study. The device had been previously calibrated against a local monitoring station (Flemish Environmental Agency, Borgerhout, Antwerp). The PM concentrations were measured both outside, at the entrance of the hospital, and inside, in the waiting room.

##### Modeled PM_10_

We calculated the regional background level of PM_10_ (previous day, week, month, 3 months, and 6 months and annual average) for each participant’s home address using a kriging interpolation method (Janssen et al. 2008). This model provides interpolated PM_10_ values from the Belgian telemetric air quality networks in 4 × 4 km grids. The interpolation was based on a detrended kriging interpolation model that uses land cover data obtained from satellite images (Corine land cover data set; European Environment Agency 2000).

#### Internal PM: carbon load of airway macrophages obtained by induced sputum

**Figure 2:**
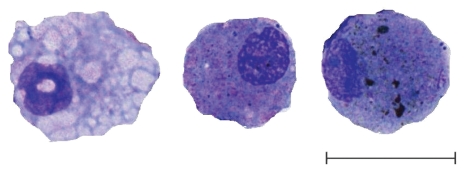
Airway macrophages with no (left), medium (middle), and high (right) carbon load. Airway macrophages were obtained by induced sputum, stained with Diff-Quik, and viewed with light microscopy. The area occupied by carbon in 50 randomly selected airway macrophages was determined by means of image analysis, and the median area (μm^2^) per cell was calculated. Bar = 20 μm.

To induce sputum, nebulized saline (NaCl 3%, 4%, or 5%) was administered through an ultrasonic nebulizer (Ultra-NebTm2000 model 200HI; DeVilbiss Healthcare, Somerset, PA, USA) in one, two, or three 7-min inhalation periods. Patients were pretreated with an inhaled β_2_-agonist (200 μg salbutamol). Pulmonary function was measured before each inhalation period for the detection of clinically significant bronchoconstriction (Paggiaro et al. 2002). To isolate airway macrophages, induced sputum was processed according to a standard technique (Pizzichini et al. 1996). Dithiothreitol (Sigma Aldrich, St. Louis, MO, USA) was used as a mucolytic agent, and airway cells were cytocentrifuged (Cytospin; Shandon Scientific, Techgen, Zellik, Belgium) onto glass slides and stained with Diff-Quick (Medion Diagnostics, Düdingen, Germany). Sputum supernatants were kept at − 80°C for future analysis. Airway macrophages (Figure 2) were visualized by light microscopy (AxioPlan 2 Imaging; Zeiss, Zaventem, Belgium). Each airway macrophage was initially processed using Paint Shop software (version 5.1; Microsoft, Zaventem, Belgium). First, the nucleus was removed from the image. Then Scion image software (Scion Corporation, Frederick, MD, USA) was used to calculate the carbon load of airway macrophages, which was defined as the median area (square micrometers) occupied by carbon, in 50 randomly selected macrophages per patient (Kulkarni et al. 2005, 2006).

### Clinical Measurements

#### Blood collection and analysis

A nonfasting blood sample was collected in an EDTA tube and in a tube containing 0.129 M (3.8%) sodium citrate for platelet function analysis and for differential cell counts, respectively. Blood cell counts (including platelet counts) and differential leukocyte counts were determined using an automated cell counter with flow differential (Cell Dyn 3500; Abbott Diagnostics, Abbott Park, IL, USA). Blood glucose levels and glycated hemoglobin were measured according to standard clinical procedures. Plasma samples were kept frozen at − 80°C for future analysis.

#### Platelet function analyzer

Platelet function was assessed with the PFA-100 platelet function analyzer (Siemens Healthcare Diagnostics, Deerfield, IL, USA). The PFA-100 test cartridge consists of a capillary, a blood sample reservoir, and a membrane coated with collagen/epinephrine with a central aperture. Whole blood is aspirated through the capillary and the aperture, thus exposing platelets to high shear rates (5,000/sec) and to collagen and epinephrine, causing platelet activation. A platelet thrombus forms at the aperture, thus gradually diminishing and finally arresting blood flow. The time from the start of aspiration until the aperture completely occludes, that is, the closure time, reflects platelet aggregation in a shear stress–dependent way (Kundu et al. 1995).

### Statistical Analysis

For database management and statistical analysis, we used SAS software (version 9.1; SAS Institute Inc., Cary, NC, USA). For comparison of means, medians, and proportions, we applied the Student *t*-test, Wilcoxon test, and the chi-square statistic, respectively. We investigated associations between markers of exposure (ambient PM_10_, PM_2.5_, and carbon load of airway macrophages) and different end points (platelet function, total and differential leukocyte counts, platelet count) using multiple linear regression. We report results of unadjusted analyses (in figures), results adjusted for age, and results of fully adjusted models. For fully adjusted models, covariates were identified by a stepwise regression procedure, with the *p*-values for variables to enter and to stay in the model set at 0.10. Covariates considered for entry in the model were age, sex, body mass index (BMI), socioeconomic status, outdoor temperature, time in traffic on the day of the examination, means of transportation to examination, time in hospital before blood draw, hour of blood draw, use of alcohol, exposure to environmental tobacco smoke, blood glucose level, glycated hemoglobin, menopausal status, oral contraception, use of statins, use of angiotensin-converting–enzyme (ACE) inhibitors, and use of antiplatelet medication. The possible effect modification of type of diabetes on the associations was studied. Regardless of the *p*-value, the type of diabetes was forced into the regression models. In a sensitivity analysis, we ran a model in which age, sex, BMI, and hour of blood draw were further forced into the models. Further, we calculated partial Spearman rank correlation coefficients for non-normally distributed variables. Q–Q plots of the residuals were used to test the assumptions of all linear models.

## Results

### Characteristics of study participants

We found no major differences between the total group of participants (*n* = 137) and those that had a complete set of measurements (PFA-100 and carbon load, *n* = 63) (Table 1). In those who consumed alcohol, the median alcohol consumption was 10 g/day [interquartile range (IQR), 22.5 g/day]. Forty women (63%) reported menopause, and eight (13%) used oral contraceptives. Among the men, 29 (40%) had type 1 diabetes, compared with 31 (48%) of the women. All patients with type 1 diabetes used insulin, whereas 77 persons (92%) of patients with type 2 diabetics used insulin medication. Eighty patients had important underlying cardiovascular disease. We found no significant differences in the demographic variables or in the distance from the hospital to the patient’s residence (32.7 vs. 30.4 km, *p* = 0.57) between patients for whom we obtained sufficient numbers of airway macrophages (*n* = 80) and those for whom we did not (*n* = 57). Outdoor mean ± SD PM_10_ measured at the entrance of the hospital on the day of the patient’s visit was 56.1 ± 29.0 μg/m^3^, and the average indoor PM_10_ concentration measured in the waiting room was 36.6 ± 18.4 μg/m^3^. Transportation to the hospital was by car for 87% of the patients and by public transport (bus) for 13%. The average distance from the patient’s home address to the hospital was 31.3 km (range, 0.7–139 km). The corresponding travel time was 26.2 min (range, 1–97 min).

### Carbon load of airway macrophages

The carbon load of airway macrophages did not correlate with age or BMI. We found no significant difference in carbon load of macrophages between men and women. Carbon load in macrophages was not associated with the recent outdoor or indoor PM_10_. However, persons living near a major road (< 100 m) had higher carbon load than did those living farther from a major road (0.29 vs. 0.17 μm^2^; *p* = 0.04). Each increase in modeled 6-month average PM_10_ at the participant’s residence was associated with an increase in the carbon load of airway macrophages (*r* = 0.30, *p* = 0.008), confirming that carbon load is a good marker of chronic exposure to PM.

### Platelet function

**Figure 3:**
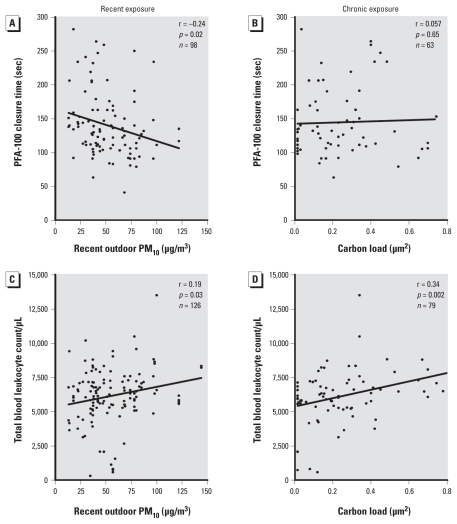
Platelet function and blood leukocytes. (*A*, *C*) Pearson correlations between recent exposure (PM_10_ measured at the study site 2 hr before clinical examination) and platelet function (*A*) or blood leukocyte count (*C*). (*B, D*) Spearman rank correlations between chronic exposure (as assessed by the carbon load of airway macrophages) and platelet function (*B*) or blood leukocyte count (*D*). Platelet function was assessed by PFA-100; decreases in closure time reflect platelet activation (i.e., prothrombotic tendency).

The PFA-100 closure time was 30.4 sec [95% confidence interval (CI), 12.3–48.5, *p* = 0.001] higher in patients on antiplatelet therapy than in those not taking antiplatelet medication (*n* = 55). None of the other studied potential covariates, including hour of blood draw, number of platelets, outdoor temperature, and travel time to the hospital, entered the stepwise regression model. Both before adjustment (Figure 3) and after adjustment (Table 2) for the use of antiplatelet medication, the closure time was inversely associated with the recent outdoor PM measured 2 hr before the examination, but not with indexes of chronic exposure. The interaction terms between use of antiplatelet medication and exposure to PM did not reach statistical significance (*p* ≥ 0.17). We observed no association between closure time and carbon load (chronic exposure). In a model combining recent exposure to PM_10_ and carbon load of airway macrophages (Table 3), the recent exposure remained negatively associated with the closure time. Forcing age, sex, BMI, and hour of blood draw into the stepwise regression models did not alter the reported findings significantly.

### Total and differential blood leukocyte counts

In a stepwise multiple regression, the number of blood leukocytes was significantly higher in persons with type 2 diabetes than in those with type 1 diabetes (767/μL; 95% CI, 77–1,456; *p* = 0.03) and increased with blood glucose (4.5/μL per mg/dL glucose; 95% CI, − 0.3 to 9.4; *p* = 0.07). Both before adjustment (Figure 3) and after adjustment (Table 2) for these covariates, the number of leukocytes correlated positively both with recent exposure and with carbon load of airway macrophages. Even in a model that combined recent exposure to PM_10_ and chronic exposure (Table 3), as assessed by the carbon load of macrophages, the chronic exposure remained positively associated with the total number of leukocytes. The blood lymphocyte count showed a stronger association with the carbon load of airway macrophages than with recent exposure to PM_10_, whereas blood neutrophils were only marginally associated with the carbon load but significantly with recent exposure (Table 2). This was also the case in the combined analysis (Table 3). We observed no significant changes in blood eosinophils and monocytes (data not shown).

In further analyses, we studied the associations between platelet function and total blood leukocyte count. Number of leukocytes was not associated with platelet function, even not after adjusting for the carbon load of airway macrophages. Forcing age, sex, BMI, and hour of blood draw into the stepwise regression models did not alter the reported findings significantly.

### Blood platelet count

We found no association between blood platelets and markers of recent exposure to PM (Table 2). The carbon load was marginally associated with the number of blood platelets (Table 2). In a model (Table 3) with both recent (PM_10_) and chronic exposure (carbon load), only the chronic exposure was associated with number of blood platelets. Forcing age, sex, BMI, and hour of blood draw into the stepwise regression models did not alter the reported findings significantly.

### Sensitivity analyses

Calculation of partial Spearman rank correlation coefficients for non-normally distributed variables confirmed our results (data not shown).

We studied possible effect modification of type of diabetes on the associations. The interaction term did not reach statistical significance in any of the models (*p* > 0.20).

Models in which we replaced the carbon load of airway macrophages with the modeled 6-month average PM air pollution near the patient’s home (4 × 4 km grid) showed no significant correlation with the studied effect parameters.

## Discussion

We observed that PM exposure appears to have a rapid prothrombotic effect on platelet function. Recent and chronic exposures to PM were associated with markers of systemic inflammation, seen as an increase in blood leukocyte counts. However, we found no association between the observed prothrombotic effect and markers of systemic inflammation. Currently, it is well recognized that thrombosis underlies most acute complications of atherosclerosis, such as acute myocardial infarction. Peters et al. (2001) showed that exposure to elevated concentrations of fine PM (PM_2.5_) for as little as 2 hr increases the risk of myocardial infarction. Long-term exposure to PM has also been suggested to play a role in the underlying pathologic process, atherosclerosis (Hoffmann et al. 2007; Künzli et al. 2005; Sun et al. 2005). The purpose of our study was not to show that persons with diabetes are more susceptible to the effects of PM air pollution, but to verify the hypothesis that PM causes prothrombotic changes in these presumably more susceptible subjects, possibly via systemic inflammation. Therefore, in the present study we combined personal markers of recent exposure (PM measured at the study site) and chronic exposure to PM as assessed by the carbon load of airway macrophages (Kulkarni et al. 2006).

Platelet activation, measured *ex vivo* with the PFA, allows a quantitative measure of platelet aggregation as the time required to close a small aperture in a biological active membrane by relevant stimuli. The average PFA closure time in our well-controlled diabetic population was comparable with the closure time in healthy subjects reported in literature (Homoncik et al. 2000; Seyfert et al. 2007). Our study shows that the closure time correlated inversely with the ambient PM air pollution concentration, measured 2 hr before the blood collection. Previously, we showed in an experimental study that the intratracheal instillation of diesel exhaust particles (DEP) in hamsters caused platelet activation within 1 hr and a dose-dependent enhanced arterial or venous thrombus (Nemmar et al. 2003a). Recently, Lucking et al. (2008) showed in a controlled exposure experiment an association between enhanced thrombus formation *ex vivo* and inhalation of DEP 2 hr after exposure. The clinical significance of the association we observed between platelet activation measured *ex vivo* and air pollution stems from prospective observations that a shorter closure time of the PFA-100 device predicts recurrent ischemic events in patients who underwent a percutaneous coronary intervention (Gianetti et al. 2006). In our study, an IQR increase of 39.2 μg/m^3^ in PM_10_ was associated with a decrease in the PFA closure time of 25 sec. If we compare this with the average effect of antiplatelet medication, it appears that the magnitude of the pollution effect (− 25 sec) is about two-thirds (in the opposite direction) of that caused by the antiplatelet medication (36 sec). Intake of a daily dose of 75 mg aspirin during 2 weeks caused an increase in the median PFA closure time of 30 sec in 10 healthy individuals (Ahmed et al. 2009). Similarly, in a population of 34 patients with type 2 diabetes, the mean PFA closure time significantly increased by 57 sec after daily intake of 100 mg aspirin during 1 week (Abaci et al. 2005).

We also documented systemic inflammatory effects because we found a positive association between the number of blood leukocytes and both recent and chronic exposure to PM air pollution. Mukae et al. (2001) showed in rabbits that repeated exposure to ambient PM_10_ caused an accelerated release of immature polymorphonuclear leukocytes from the bone marrow. The magnitude of the stimulation of the bone marrow by PM_10_ was related to the quantity of particles phagocytosed by alveolar macrophages. Our findings that the carbon load of airway macrophages is associated with increases in blood leukocytes are in line with these experimental findings. Long-term changes in leukocyte counts in association with PM have also been investigated in human epidemiologic studies. Recent observations of the Third National Health and Nutrition Examination Survey showed a positive association between chronic (1 year) exposure to PM_10_ and blood leukocyte counts (Chen and Schwartz 2008). A study of 39 Japanese men traveling to Antarctica, an area with low exposure to PM, showed a 17% decrease in leukocyte counts (Sakai et al. 2004).

This study is novel in that it suggests that persons with diabetes, a condition associated with chronic inflammation, may have a short-term inflammatory response to recent PM air pollution, in addition to the effect of chronic exposure as assessed by the carbon load of airway macrophages. In persons without diabetes, studies looking for short-term changes in leukocyte counts in relation to air pollution have given inconclusive results. Two studies that reported significant results for leukocyte counts had opposite findings (Ghio et al. 2003; Schwartz 2001), and other studies reported null associations (Holgate et al. 2003; Pope et al. 2004; Seaton et al. 1999).

It has been shown that chronic inflammation is involved in the development of atherosclerosis (Ross 1999). Chronic exposure to PM leading to systemic inflammation might therefore also play a role in the development of atherosclerosis. Exposing apolipoprotein E–null mice for 6 months to an equivalent concentration of 15.2 μg/m^3^ PM_2.5_ over a lifetime, Sun et al. (2005) found that transverse sections of abdominal aorta increased in percentage plaque area compared with mice exposed to filtered air. Suwa et al. (2002) showed in rabbits that repeated exposure to PM_10_ was associated with both systemic inflammation and the progression of the atherosclerotic process, the extent of which correlated with the extent of PM_10_ phagocytosed by alveolar macrophages. Chronic inflammation is more prominent in type 2 diabetes than in persons with type 1 diabetes. However, we did not find evidence of a higher sensitivity to air pollution–induced effects on platelet function or leukocyte distribution in persons with type 2 diabetes compared with their type 1 counterparts. O’Neill et al. (2005) found a stronger association between endothelial function and PM air pollution in type 2 compared with type 1 diabetes. In our study, patients had well-controlled glycated hemoglobin levels, which averaged 7.4%. Moreover, insulin use in persons with type 2 diabetes was high.

We did not observe a link between leukocyte counts and platelet activation. This suggests that PM may have effects on platelet function independently of systemic inflammation. In experimental conditions using DEP, Nemmar et al. (2003b) showed a prothrombotic tendency and activation of circulating blood platelets, as well as lung inflammation, which persisted up to 24 hr after instillation of DEP in hamsters. However, the prothrombotic tendency observed 1 hr after DEP exposure did not appear to correlate with pulmonary inflammation (Nemmar et al. 2003b).

Our study has limitations. Observational studies do not prove causality, even when exposure is measured on an individual level. Recent exposure to PM was based on measurements at the hospital. We modeled PM data but no personalized exposure measurements 24 hr before blood draw. The carbon load of airway macrophages may not reflect the load of carbon in more distal alveolar cells, because sputum induction samples macrophages from the larger airways (Alexis et al. 2001). In adults, however, the distribution of PM from the environment in bronchial macrophages is nearly identical to that in alveolar macrophages (Fireman et al. 2004; Lay et al. 1998). Our limited success rate of 58% for sputum induction may have introduced bias, but we found no differences in any other measured variables between those from whom sputum was induced successfully and those from whom it was not. Because the method used to assess the carbon load of 50 airway macrophages per persons is labor intensive, our study sample did not include a very large number of participants, but this group size has been shown to be relevant when using this surrogate of personal exposure to PM (Kulkarni et al. 2006).

The carbon load in airway macrophages was associated with modeled 6-month average PM_10_ exposure at the patient’s home. However, the blood leukocyte count was not significantly associated with the modeled 6-month average PM_10_ concentration, although it was with the carbon load of airway macrophages. This suggests that the latter biomarker of chronic exposure might be a better reflection of personal exposure to PM. For the modeled previous day, week, month, 3-month, and annual average PM_10_ at the patient’s residence, we found no correlations (*p* ≥ 0.15) with the carbon load of airway macrophages.

Our findings have important implications for understanding the biological mechanisms of air pollution on cardiovascular health and its clinical relevance, because both a prothrombotic tendency and systemic inflammation play an important role in atherosclerosis and cardiovascular disease. The clinical relevance of our findings in persons with diabetes is evident from the observation that a realistic increase in recent PM air pollution exposure was associated with a change in platelet function toward a greater prothrombotic tendency. The magnitude of this change was about two-thirds (in opposite direction) of the average effect of antiplatelet medication.

## Figures and Tables

**Table 1:** Patient characteristics.

Characteristic	Total group (*n* = 137)	Group with carbon load and PFA-100 (*n* = 63)
Anthropometrics		
Sex (female)	64 (47)	35 (56)
Age (years)	54.7± 14.4	51.5 ± 14.5
BMI (kg/m^2^)	28.4 ± 5.4	28.0 ± 5.2
Type 1 diabetes	60 (44)	29 (46)
Blood glucose (mg/dL)	145 ± 71.9	151.7 ± 72.7
Glycated hemoglobin (%)	7.4 ± 1.0	7.4 ± 1.1
Lifestyle		
Regular alcohol use	36 (26)	18 (29)
Exposure to environmental tobacco smoke	27 (20)	10 (16)
Socioeconomic status		
Low	79 (58)	32 (51)
Middle	44 (32)	21 (33)
High	14 (10)	10 (16)
Use of medication		
Antiplatelet medication^a^	82 (60)	34 (54)
Statins	88 (64)	37 (59)
ACE inhibitor	60 (44)	21 (33)
Insulin	130 (95)	61 (97)
Antidiabetic medication	50 (36)	21 (33)
Exposure markers		
Recent (2 hr) outdoor PM_2.5_ (μg/m^3^)	25.1 ± 18.4	23.7 ± 16.0
Recent (2 hr) outdoor PM_10_ (μg/m^3^)	56.1 ± 29.0	53.2 ± 24.8
Six-month average modeled PM_10_ (μg/m^3^)	25.3 ± 3.7	25.4 ± 3.9
Carbon load in airway macrophages (μm^2^)	0.19 (0.09–0.34)^b^	0.20 (0.10–0.34)
End points		
PFA-100 closure time (sec)	140 ± 47.9^c^	144 ± 50.4
Total blood leukocytes/μL	6,152 ± 2,027^d^	6,010 ± 2,134
Blood neutrophils/μL	3,826 ± 1,378^d^	3,706 ± 1,371
Blood eosinophils/μL	164 ± 138^d^	169 ± 163
Blood monocytes/μL	420 ± 157^d^	417 ± 151
Blood lymphocytes/μL	1,170 ± 739^d^	1,783 ± 697
Blood platelets ×10^3^/μL	232 ± 62.4^d^	232 ± 60

Values are number (%) or arithmetic mean ± SD, except for the carbon load, which was not normally distributed, for which the median (IQR) is given.

a Antiplatelet medication included acetylsalicylic acid, clopidogrel, ticlopidine, or dipyridamole.

b Data available for 80 subjects.

c Data available for 98 subjects.

d Data available for 126 subjects.

**Table 2:** Change in platelet function and in total or differential blood leukocyte counts and platelet count for an IQR increase in recent outdoor PM_2.5_ or PM_10_ concentrations or in carbon load of airway macrophages (separate analysis).

End point	Exposure marker, IQR	Age-adjusted difference (95% CI)	*p*-Value	Adjusted^a^ difference (95% CI)	*p*-Value
PFA-100 closure time (sec)	PM_2.5_, 22.3 μg/m^3^	− 12.4 (− 25.8 to 1.0)	0.07	− 16.3 (− 29.0 to − 3.7)	0.01
	PM_10_, 39.2 μg/m^3^	− 19.0 (− 34.1 to − 3.8)	0.02	− 21.1 (− 35.3 to − 6.8)	0.005
	Carbon load, 0.25 μm^2^	3.2 (− 12.7 to 19.1)	0.69	3.8 (− 11.8 to 19.5)	0.63

Total blood leukocyte count/μL	PM_2.5_, 22.3 μg/m^3^	544 (104 to 983)	0.02	451 (40.5 to 860)	0.03
	PM_10_, 39.2 μg/m^3^	577 (79.8 to 1,075)	0.02	512 (45.2 to 979)	0.03
	Carbon load, 0.25 μm^2^	760 (290 to 1,230)	0.002	687 (224 to 1,150)	0.005

Neutrophils/μL	PM_2.5_, 22.3 μg/m^3^	318 (18.4 to 618)	0.04	278 (− 2.25 to 558)	0.05
	PM_10_, 39.2 μg/m^3^	378 (40.4 to 716)	0.03	360 (42.8 to 668)	0.03
	Carbon load, 0.25 μm^2^	353 (33.1 to 673)	0.03	294 (− 20.0 to 609)	0.07

Lymphocytes/μL	PM_2.5_, 22.3 μg/m^3^	196 (35.7 to 356)	0.02	147 (− 1.0 to 294)	0.05
	PM_10_, 39.2 μg/m^3^	160 (− 23.5 to 343)	0.09	110 (− 60.1 to 280)	0.21
	Carbon load, 0.25 μm^2^	199 (46.5 to 351)	0.01	221 (72.2 to 370)	0.005

Platelets ×10^3^/μL	PM_2.5_, 22.3 μg/m^3^	− 2.7 (− 16.5 to 11.0)	0.70	− 4.3 (− 17.3 to 8.8)	0.52
	PM_10_, 39.2 μg/m^3^	0.7 (− 14.9 to 16.2)	0.93	− 0.7 (− 15.8 to 14.3)	0.92
	Carbon load, 0.25 μm^2^	13.0 (− 1.6 to 27.5)	0.09	14.1 (− 0.3 to 28.5)	0.06

Differences calculated for an IQR increase in exposure variables,

a Adjusted for significant (*p* < 0.10) covariates (see text) identified by stepwise regression. Covariates considered for entry in the model were age, sex, BMI, socioeconomic status, outdoor temperature, time in traffic on day of exam, means of transportation to the exam, time in hospital before blood draw, hour of blood draw, use of alcohol, exposure to environmental tobacco smoke, blood glucose level, glycated hemoglobin, menopausal status, oral contraception, use of statins, use of ACE inhibitors, and use of antiplatelet medication. Type of diabetes was forced into all models.

**Table 3:** Change in platelet function and in total or differential blood leukocyte counts and platelet count for an IQR increase in recent outdoor PM_10_ concentrations and in carbon load of airway macrophages (combined analysis).

End point	Exposure marker, IQR	Age-adjusted difference (95% CI)	*p*-Value	Difference adjusted for significant covariates (95% CI)^a^	*p*-Value
PFA-100 closure time (sec)	PM_10_, 39.2 μg/m^3^	− 18.8 (− 38.4 to 0.75)	0.06	− 25.4 (− 44.4 to − 6.3)	0.01
	Carbon load, 0.25 μm^2^	2.1 (− 13.5 to 17.7)	0.79	2.8 (− 12.2 to 17.7)	0.72

Total blood leukocyte count/μL	PM_10_, 39.2 μg/m^3^	770 (249 to 1291)	0.005	737 (239 to 1,236)	0.005
	Carbon load, 0.25 μm^2^	806 (356 to 1,255)	0.0008	747 (303 to 1,190)	0.002

Neutrophils/μL	PM_10_, 39.2 μg/m^3^	462 (102 to 821)	0.01	451 (109 to 793)	0.01
	Carbon load, 0.25 μm^2^	381 (70.4 to 691)	0.02	331 (26.5 to 635)	0.04

Lymphocytes/μL	PM_10_, 39.2 μg/m^3^	245 (75.4 to 414)	0.006	220 (58.0 to 382)	0.01
	Carbon load, 0.25 μm^2^	213 (67.3 to 360)	0.006	242 (97.9 to 386)	0.002

Platelets ×10^3^/μL	PM_10_, 39.2 μg/m^3^	9.2 (− 7.7 to 26.1)	0.29	8.8 (− 7.6 to 25.1)	0.30
	Carbon load, 0.25 μm^2^	13.5 (− 1.1 to 28.1)	0.07	14.7 (0.29 to 29.2)	0.05

a Adjusted for significant (*p* < 0.10) covariates (see text) identified by stepwise regression. Covariates considered for entry in the model were age, sex, BMI, socioeconomic status, outdoor temperature, time in traffic on day of exam, means of transportation to exam, time in hospital before blood draw, hour of blood draw, use of alcohol, exposure to environmental tobacco smoke, blood glucose level, glycated hemoglobin, menopausal status, oral contraception, use of statins, use of ACE inhibitors, and use of antiplatelet medication. Type of diabetes was forced into all models.
